# Supplementary material for: Delivery of community-centred public mental health interventions in diverse areas in England: a mapping study protocol
Source: BMJ Open. 2020 Jul 13;10(7):e037631. doi: 10.1136/bmjopen-2020-037631 (PMC7359052; doi:10.1136/bmjopen-2020-037631)
Supplement: Author's manuscript [file bmjopen-2020-037631.draft_revisions.pdf]

BMJ Open

BMJ Open is committed to open peer review. As part of this commitment we make the peer review history of every article we publish publicly available.

When an article is published we post the peer reviewers' comments and the authors' responses online. We also post the versions of the paper that were used during peer review. These are the versions that the peer review comments apply to.

The versions of the paper that follow are the versions that were submitted during the peer review process. They are not the versions of record or the final published versions. They should not be cited or distributed as the published version of this manuscript.

BMJ Open is an open access journal and the full, final, typeset and author-corrected version of record of the manuscript is available on our site with no access controls, subscription charges or pay-per-view fees (http://bmjopen.bmj.com).

If you have any questions on BMJ Open's open peer review process please email info.bmjopen@bmj.com

BMJ Open

Mapping the delivery of community-centred public mental health interventions in diverse areas in England: study protocol.

Journal:	*BMJ Open*
Manuscript ID	bmjopen-2020-037631
Article Type:	Protocol
Date Submitted by the Author:	10-Feb-2020
Complete List of Authors:	Duncan, Fiona; Durham University, Sport and Exercise Sciences<br>McGrath, Mike; UCL, Division of Psychiatry; UCL, Department of Primary Care and Population Health<br>Baskin, Cleo; Imperial College London, Department of Primary Care and Public Health<br>Osborn, David; UCL, Division of Psychiatry<br>Dykxhoorn, Jen; UCL, Division of Psychiatry; UCL, Department of Primary Care and Population Health<br>Kaner, Eileen; Newcastle University, Institute of Health and Society<br>Gnani, Shamini; Imperial College London, Department of Primary Care and Public Health<br>LaFortune, Louise; University of Cambridge, Institute of Public Health<br>Lee, Caroline; University of Cambridge, Institute of Public Health<br>Walters, Kate; University College London, Primary Care and Population Health<br>Kirkbride, James; University College London, Division of Psychiatry<br>Fischer, Laura; McPin Foundation<br>Jones, Oli; McPin Foundation<br>Pinfold, Vanessa; McPin Foundation,<br>Stansfield, J; Public Health England, Health Improvement Directorate<br>Oliver, Emily; Durham University, Department of Sport and Exercise Sciences
Keywords:	PUBLIC HEALTH, MENTAL HEALTH, Health policy < HEALTH SERVICES ADMINISTRATION & MANAGEMENT, Adult psychiatry < PSYCHIATRY

SCHOLARONE™
Manuscripts

I, the Submitting Author has the right to grant and does grant on behalf of all authors of the Work (as defined in the below author licence), an exclusive licence and/or a non-exclusive licence for contributions from authors who are: i) UK Crown employees; ii) where BMJ has agreed a CC-BY licence shall apply, and/or iii) in accordance with the terms applicable for US Federal Government officers or employees acting as part of their official duties; on a worldwide, perpetual, irrevocable, royalty-free basis to BMJ Publishing Group Ltd ("BMJ") its licensees and where the relevant Journal is co-owned by BMJ to the co-owners of the Journal, to publish the Work in this journal and any other BMJ products and to exploit all rights, as set out in our licence.

The Submitting Author accepts and understands that any supply made under these terms is made by BMJ to the Submitting Author unless you are acting as an employee on behalf of your employer or a postgraduate student of an affiliated institution which is paying any applicable article publishing charge ("APC") for Open Access articles. Where the Submitting Author wishes to make the Work available on an Open Access basis (and intends to pay the relevant APC), the terms of reuse of such Open Access shall be governed by a Creative Commons licence – details of these licences and which Creative Commons licence will apply to this Work are set out in our licence referred to above.

Other than as permitted in any relevant BMJ Author's Self Archiving Policies, I confirm this Work has not been accepted for publication elsewhere, is not being considered for publication elsewhere and does not duplicate material already published. I confirm all authors consent to publication of this Work and authorise the granting of this licence.

For peer review only

Mapping the delivery of community-centred public mental health interventions in diverse areas in England: study protocol.

Duncan F.[1], McGrath M.[2], Baskin C.[4], Osborn D.[2,7], Dykxhoorn J.,[2,3] Kaner E.[5], Gnani S.[4], Lafortune L.[6], Lee C.[6], Walters K.[3], Kirkbride J.B.[2] Fischer L.E.[8], Jones O.[8], Pinfold V.[8], Stansfield J.[9], Oliver E.[1], on behalf of the NIHR SPHR Public Mental Health Programme.

[1]Department of Sport and Exercise Sciences, Durham University, 42 Old Elvet, Durham, DH1 3HN,

[2]Division of Psychiatry, UCL, Maple House, 149 Tottenham Court Road, London, W1T 7BN.

[3]Department of Primary Care and Population Health, UCL, Royal Free, Rowland Hill Street London, NW3 2PF.

[4] Department of Primary Care and Public Health, School of Public Health, Imperial College London, Reynolds Building, St Dunstan's Road, London, W6 8RP.

[5]Institute of Health and Society, Baddiley-Clark Building, Newcastle University, Newcastle upon Tyne, NE2 4AX.

[6]Cambridge Institute of Public Health, Cambridge University, Forvie Site, Robinson Way, Cambridge, CB2 0SR.

[7]Camden and Islington NHS Foundation Trust, London, NW1 OPE

[8]McPin Foundation, 7-14 Great Dover Street, London, SE1 4YR

[9]Health Improvement Directorate, Public Health England.

Corresponding author: Fiona Duncan, Department of Sport and Exercise Sciences, Durham University, 42 Old Elvet, Durham, DH1 3HN, 0191 334 4676, fiona.h.duncan@durham.ac.uk

Keywords: public mental health, community-centred interventions, intervention mapping.

Word Count: 2453

For peer review only - http://bmjopen.bmj.com/site/about/guidelines.xhtml

Abstract

Background

Public Mental Health (PMH) is a global challenge and a UK priority area for action. However, to progress, practitioners require a stronger evidence base regarding the effectiveness of approaches, particularly regarding promotion and prevention through community-centred interventions. In addition, policy-makers need to understand what is being delivered, particularly in areas of high need, to identify promising practices or gaps in PMH provision. Thirdly, the public need better information regarding what approaches and services are available to them. We report a protocol designed to 1) identify the types of community-centred interventions employed in purposively selected diverse geographical areas of England to improve PMH outcomes and 2) describe the type, target population, content and outcome measures of each intervention.

Methods and analysis

Five local authority areas of England were selected based on either high social deprivation or differing ethnic population statistics and geographical locations. Community-centred interventions in each area will be identified through: (i) desk-based data capture from standardised searches of publicly-available information (e.g. policy, strategy, intervention advertising), (ii) established professional networks and service contacts, (iii) chain-referral sampling of individuals involved in local MHP and prevention and (iv) peer researchers, who will use their personal experience and local knowledge to help identify potentially relevant organisations. Data of the key features of the interventions will be extracted from individuals either by structured interview or by electronic questionnaire with information regarding the intervention(s) of which they have knowledge. Initial data analysis will involve tabulating descriptive information. A descriptive comparison will be made between selected geographical areas.

Ethics and Dissemination

For peer review only

Ethical approval was obtained Durham University's Department of Sport and Exercise Sciences Research Ethics Committee. We plan to disseminate our findings at conferences and through peer-reviewed journals. We also plan to disseminate to the public and intervention providers through social media and/or newsletters.

Article Summary

Strengths and limitations of this study

- Multiple sources are used to collect data.
- Protocol has been piloted in two areas and refined.
- A range of geographical areas of England have been included.
- It cannot be guaranteed that key individuals will be able to provide accurate information or that websites will be up-to-date.
- Only services that explicitly aim to improve the mental health of adults have been included (services aimed at only children and adolescents have been excluded).

Background

For peer review only

Public mental health (PMH) or population mental health is an emerging field and consequently there is still a lack of consensus regarding its definition and key components [1]. The World Health Organisation's (WHO) [2] PMH approach emphasises the distinction between mental health and mental illness, stating that mental health should be considered to be more than the absence of illness, and therefore conceptualisations of PMH should include mental health promotion as well as mental illness prevention. Mental health promotion involves encouraging good mental health, reducing inequalities, building social capital, enhancing quality of life and enabling optimal psychological and psychophysiological development throughout the life course. Mental illness prevention involves reducing the incidence, prevalence and recurrence of mental disorders, as well as reducing the risk factors and the impact of mental illness on the affected person [2]. Consequently, WHO recommended in their mental health action plan several actions that focused on the promotion of positive mental health and emotional well-being as well as the prevention of mental health issues [3]. Consistent with this, Public Health England (PHE), a UK Government agency, also conceptualises PMH as consisting of the promotion of good mental health and the prevention of mental health problems and suicide [4]. A key aspect of PHE's approach to PMH is the recognition that mental health is a complex determinant of overall health and wellbeing and therefore actions that aim to minimise risk factors and enhance protective factors of mental health should be incorporated into a wide range of public health policies [4]. In line with the viewpoint of WHO and PHE, the conceptualisation of PMH that will be adopted for this protocol includes adverse and positive mental health [5].

For peer review only

Improving PMH is important as each year, in England, almost a quarter of adults experience at least one mental health problem [6] and 17% experience a sub-threshold mental health problem [6]. As well as the impact on the individual, this creates a substantial economic and social burden for the country as mental health problems have been reported to be associated with increased risk of physical illness [7,8] such as coronary heart disease [9], increased mortality [10], higher rates of health risk behaviours such as smoking [11], reduced life expectancy [12], work absence [13], unemployment [14], homelessness [15] and reduced quality of life [16]. Conversely, good mental wellbeing has been

For peer review only - http://bmjopen.bmj.com/site/about/guidelines.xhtml

reported to be associated with reduced mental health problems [17] and suicide [18], reduced heart disease [19], reduced mortality and increased life expectancy [20], improved recovery from physical illness [21], increased physical activity [22], reduced absenteeism [23] increased productivity at work, higher income and stronger social relationships [17].

Despite the high prevalence of mental health problems there has been a chronic underinvestment in mental health prevention across the English National Health Service (NHS) in recent years [24] and provision of interventions and services that support PMH in local areas has been badly affected by austerity measures introduced by the UK government in 2010 [25,26,27]. There is ongoing concern about how to promote PMH in areas experiencing deprivation, in particular [25,26,27].

A range of policy documents and publications have highlighted the importance of addressing the modifiable determinants of PMH within communities [15, 27, 28, 29]. These modifiable potential factors include both acute and chronic determinants such as economic disadvantage [6, 30], debt and financial difficulties [31,32,33,34], unemployment [14], social isolation and loneliness [14], intimate partner violence [35], sedentary lifestyles [36,37], fuel poverty [38], food insecurity [39,40], homelessness [41] and belonging to an ethnic minority group and other groups that experience stigma/marginalisation [14].

If causal, such a wide range of inter-related modifiable factors suggests that a multitude of approaches may be needed to improve mental health. However, the current evidence base for the effectiveness and cost-effectiveness of community-centred PMH interventions for adults is limited [42]. Promising examples of PMH interventions include workplace-based stress management interventions [43], community interventions for loneliness in older adults [44,45], and welfare benefits and debt advice interventions based in GP practices [46]. While these show promise to improve mental health and wellbeing outcomes, the precise ‘active ingredients’ of these interventions need further evidence [47]. Further, in order to progress the provision of PMH services and approaches we need to understand what is currently being delivered in local areas, so that the assets and capabilities available in different

For peer review only

For peer review only - http://bmjopen.bmj.com/site/about/guidelines.xhtml

communities addressing modifiable determinants of mental health are accurately mapped. Specifically, we need to identify any promising practice, learning from existing (successful and unsuccessful) practice, inconsistencies, variations or gaps in service delivery and what evidence exists concerning the effect they have on meeting population needs. As part of a larger National Institute of Health Research (NIHR) funded programme through the School for Public Health Research (SPHR), the present study protocol aims to:

1) identify the types of community-centred interventions employed in purposively selected diverse geographical areas of England to improve PMH outcomes and
2) describe the type, target population, risk/protective factor, content, reach, effectiveness, cost-effectiveness and intended outcome measures of each intervention.

Methods

Selected areas of England

The following local authority areas of England have been selected: Redcar and Cleveland (North East), Cambridgeshire and Peterborough (East), Blackburn (North West) and Camden and Islington, Hammersmith and Fulham (Greater London).

The process of selecting these areas was as follows: 1) a longlist of candidate local authority areas in the North East, North West, London and East of England was created to ensure that a wide geographic spread of areas were represented. 2) A range of statistics from the Office of National Statistics (ONS) annual population survey regarding these local authority areas were examined, including statistics on deprivation. 3) The final areas were chosen as they have diverse social deprivation and/or ethnic populations, were proximal to research group institutions associated with the NIHR SPHR and therefore research group members could use established contacts with relevant local authority personnel to maximise response rates.

For peer review only

For peer review only - http://bmjopen.bmj.com/site/about/guidelines.xhtml

This protocol was piloted by mapping two of these selected areas (Redcar and Cleveland, Camden and Islington) between August 2019 and January 2020. We plan to have completed the mapping of the remaining areas by the end of May 2020.

Inclusion and Exclusion criteria

The following inclusion criteria will be applied to identify potential PMH interventions:

- Any intervention which explicitly aims to improve adult mental health and wellbeing. This might include support, individual advice giving, signposting, activity groups or referral to community services. These programmes and interventions can be aimed at the general population or targeted at a specific sub-group i.e. older people, particular ethnic minorities, people who have job insecurity/debt/housing problems. Interventions delivered by individuals within the community (e.g. workshops, support groups) will be included.
- Any intervention which is delivered in, or available to residents of, the target local authority area, even if it is funded, delivered or managed within another local authority area or larger geographical area. Online and digital interventions will be included.
- Any intervention delivered by the government, the local authority, third sector organisations, social enterprises, the NHS (if non-clinical), or located in primary care settings (if non-clinical).
- Programmes and interventions that are available to local residents during the data collection period.

An intervention will be excluded if, it is:

- A clinical mental health intervention or an intervention that is primarily aimed at people identified with a diagnosed or suspected mental health condition.
- A wholly (for-profit) private sector funded intervention.

For peer review only - http://bmjopen.bmj.com/site/about/guidelines.xhtml

- An intervention which is exclusively aimed at children or adolescents, as this mapping exercise is focused on services for adults.
- Improving mental health is not one of the intervention's explicit aims (whether the aim is stated in writing or verbally by an intervention provider).

Data Collection

Two types of information will be sought during the mapping exercise: 1) identification of relevant interventions; 2) data on the key features of the identified services.

1) Identification of interventions. Much information about local services is unpublished and available information online can rapidly become out of date. Therefore, we will employ a range of approaches to create a list of interventions as exhaustive as possible, bringing together information from four sources: i) Desk-based searches of available information resources. Local authority, relevant third sector, NHS and Clinical Commissioning Group websites will be searched for key policy documents, strategies, intervention advertising or guides to local services (Table 1). General internet searches using the following key search terms will also be carried out: public mental health interventions, community development, community capacity building, community activities, anxiety/depression/stress courses/groups/workshops, wellbeing workshops/courses/groups/activities/sessions, life skills courses/classes, mental health promotion, mental health prevention, suicide prevention. Social media platforms will also be searched using these terms. Potentially relevant contact details of individuals and/or organisations identified through these searches will be noted and followed-up by email or by phone for further information. ii) Requesting information through key informants in each area (suggested key informants are presented in Table 1). This will include relevant contacts from the public health department of local authorities, Clinical Commissioning Groups, voluntary sector provider organisations and signposting services (e.g. link workers or community navigators). Local authority personnel will be approached via

For peer review only - http://bmjopen.bmj.com/site/about/guidelines.xhtml

telephone and email asking for information on interventions which fit our inclusion criteria. iii) Chain-referral sampling (Snowballing). When individuals involved in local PMH promotion respond with information for the study, they will be asked if they could provide details of any other public mental health interventions or relevant organisations that they are aware of in the specified local authority area. (iv) Peer researchers working within the mental health programme of the School for Public Health Research will use their personal experience of managing poor mental health and their local knowledge to help identify potentially relevant organisations or individuals. They will also contact these organisations to obtain information about services, and follow-up with services already identified to obtain further information.

Table 1 – Key policy, strategy and signposting documents from each selected area to be searched and examples of potential key local authority departments and organisations to be approached.

**Key Documents**	**Key local authority departments**	**Key Third Sector Organisations**
• Public Mental Health strategy or action plan (e.g. prevention concordat, THRIVE, suicide prevention action plan)<br>• Clinical Commissioning Groups (CCG) Annual Report and Accounts<br>• Joint Health and Wellbeing Strategy<br>• Annual Public Health Report<br>• Joint Strategic Needs Assessment<br>• The Local Sustainability and Transformation Plan<br>• The Local Mental Health Strategy<br>• Signposting documents, websites and guides to mental health services.	• Public Health<br>• Housing<br>• Community Safety<br>• Community Health<br>• Social care<br>• Adult Education<br>• Libraries, arts and culture<br>• Leisure and recreation<br>• Neighbourhood and communities.	• MIND<br>• Citizens Advice Bureau<br>• Key Housing Associations<br>• Community Grants Organisation<br>• Job Centre Plus<br>• Age UK<br>• Age Concern<br>• Rethink Mental illness<br>• Samaritans<br>• Together<br>• CALM – Campaign against living miserably<br>• Carer's Trust

For peer review only

For peer review only - http://bmjopen.bmj.com/site/about/guidelines.xhtml

2) Data of the key features of the interventions. After identifying eligible interventions, we will extract information from websites, local authority documents and key individuals. The list of data to extract (Table 2) was adapted from the TIDieR checklist [48] to be applicable for complex public health interventions. Data will be extracted from individuals either by structured interview or an electronic questionnaire with information regarding the intervention(s) of which they have knowledge.

Table 2 – Data to be extracted about each intervention if available. (List adapted from the TIDieR checklist [48]).

1.Name of Intervention

2.Type of Intervention (Befriending; practical advice, education and advocacy; counselling; peer support; strategy or policy; activity/social group).

3.Aims and Objectives (e.g. what problem or risk factor does the intervention seek to address)

4.What are the intended primary and secondary outcomes?

5.Description of the content of the intervention (what activities, procedures, processes take place, schedule, duration and intensity).

6.Target population (who is eligible to take part)

7.Geographical area of delivery

8.Setting (e.g. community centre, sports club, home-based)

9.What facilities, infrastructure and support are required to deliver intervention?

10.How is it delivered (online, one-to-one, in groups)?

11.Who delivers the intervention (expertise, background, training)?

12.Who is the provider?

13.How is it funded?

14.What is the funding period?

15.Has the intervention been evaluated?

16. If the intervention has been evaluated, are the following data available:

- What were the headline conclusions?
- What was the methodology?
- What were the participants characteristics, reach and uptake?
- What was the reported impact on outcomes, was there any feedback from service users?
- Were there any unintended consequences?
- Did people adhere to intervention

For peer review only

For peer review only - http://bmjopen.bmj.com/site/about/guidelines.xhtml

- Were these any issues with intervention fidelity (gaps in delivery, local adaptation, modification or personalisation)?
- Is the intervention sustainable or scalable?

Data Analysis

Descriptive information for each intervention will be tabulated. Interventions will be grouped according to their intervention type (examples of potential intervention types include signposting, befriending; legal or financial practical advice, education and advocacy; counselling, peer support, strategy and policy; activity/social group, green space/area regeneration), target population and intended primary outcome (e.g. well-being or measures of mental distress). A descriptive comparison will be made between the selected geographical areas.

Patient and Public Involvement Statement

Peer researchers were recruited and coordinated by the McPin Foundation (a charity which aims to deliver more meaningful and more impactful patient and public involvement in every stage of mental health research). The peer research team was recruited to provide diverse perspectives based on their different localities, backgrounds, identities, and lived experiences of mental health challenges. The role of this team is to ensure that the research conducted in the public mental health programme is in line with the lived experience of mental health and is relevant to the challenges that people face in their daily lives.

The peer researchers have been involved with the piloting of the protocol by helping to identify potentially relevant organisations and individuals in two of the selected geographical areas that we intend to map and then contacting some of these organisations and individuals to gather information about PMH interventions they are delivering. We plan to have the peer researchers involved in a similar way when mapping the remaining selected geographical areas. They will also be involved in the analysis, write up and dissemination phases of the study. They will contribute to the writing of the study report and they will advise on the most effective approach of disseminating information to

For peer review only - http://bmjopen.bmj.com/site/about/guidelines.xhtml

community groups, voluntary sector organisations, public health practitioners and other public stakeholders and will be involved in creating the content of the dissemination. This may include producing materials such as posters and leaflets, running workshops, using social media and their hashtag #IamPublicMentalHealth, and making vlogs or writing blogs.

Ethics and Dissemination

Ethical approval was obtained from Durham University's Department of Sport and Exercise Sciences Research Ethics Committee. Application Reference: SPORT-2019-06-28T15:10:42-lxkc61. We plan to disseminate our findings at relevant conferences, meetings and through peer-reviewed journals. We will also disseminate our findings to the key personnel and organisations that participate in the study, to people who may benefit from identified interventions and the general public through social media, newsletters or local community groups. Finally, we will disseminate our findings through the SPHR's PMH virtual stakeholder network which consists of academics, voluntary sector individuals, public health practitioners, and public stakeholders.

For peer review only

Strengths and limitations of this study

Strengths

A major strength of this protocol is that it uses multiple sources to identify interventions and therefore increases the likelihood of all interventions in an area being discovered, including ones that the local authority or prominent third sector organisations are not aware of themselves. Another strength is that it has already been piloted in two of the selected areas, demonstrating its feasibility, and refinements have been made (e.g. we decided to only include interventions which were available during data collection period due to feasibility issues of collecting information about interventions that were no longer being delivered). It is also a strength that diverse geographical areas across are included in this study, increasing the generalisability of the mapping results.

Limitations

For peer review only - http://bmjopen.bmj.com/site/about/guidelines.xhtml

A potential weakness of the protocol is that there is no guarantee that key individuals who are approached for information will be willing or able to provide the most accurate and up-to-date information about interventions and services in their area. There is also the possibility that local authority and third sector websites may not be regularly updated or that there will be variations in their comprehensiveness. However, using more than one source to obtain information about each intervention should address these limitations. Another potential limitation is that we are focused on interventions and services that are explicitly aiming to improve mental health. It is possible that many services are in existence that do have a positive effect on participants' mental health but it is not their explicit aim e.g. a cycling group, a yoga course, a tennis club or a dance class. It is also a limitation that no areas in the South West of England are included in this study.

Declarations

Ethics approval and consent to participate

Ethical approval was obtained Durham University's Department of Sport and Exercise Sciences Research Ethics Committee. Application Reference: SPORT-2019-06-28T15:10:42-lxkc61

Availability of data and materials

Not applicable

Competing Interests

None

Funding

This project is funded by the National Institute for Health Research (NIHR) School for Public Health Research (SPHR) (Grant Reference Number PD-SPH-2015). The views expressed are those of the author(s) and not necessarily those of the NIHR or the Department of Health and Social Care.

Author Contributions

EO and DO are the Principal Investigators. JD is the Programme Manager. FD, MM, EO, SG, DO, KW, LL, JD, EK, LF, OJ, VP, JS were involved with designing the protocol methods. FD, MM, CB, CL. OJ and other local peer researchers will be involved with applying the protocol to collect data. JK, FD, MM, LF, VP and CB will be involved with data analysis. FD led on the writing and editing of the

For peer review only

For peer review only - http://bmjopen.bmj.com/site/about/guidelines.xhtml

manuscript. All authors contributed to the writing and editing of the protocol for publication and read and approved the final manuscript.

Acknowledgements

We would like to sincerely thank all of the local authority and third sector staff and volunteers who gave up their time to contribute to this study. We would also like to thank our peer researchers and the McPin Foundation for their invaluable help.

DO is in part supported by the National Institute for Health Research (NIHR) Collaboration for Leadership in Applied Health Research and Care (CLAHRC) North Thames at Bart's Health NHS Trust and the University College London Hospitals National Institute for Health Research Biomedical Research Centre.

The NIHR School for Public Health Research is a partnership between the Universities of Sheffield; Bristol; Cambridge; Imperial; and University College London; The London School for Hygiene and Tropical Medicine (LSHTM); LiLaC – a collaboration between the Universities of Liverpool and Lancaster; and Fuse - The Centre for Translational Research in Public Health a collaboration between Newcastle, Durham, Northumbria, Sunderland and Teesside Universities

References

1. Davies SC, Mehta N. Public mental health: evidence to policy. *World Psychiatry*. 2015;14(1):44-45.
2. World Health Organisation. Prevention of mental disorders: effective interventions and policy options. Summary Report. Geneva: World Health Organisation, 2004.
3. World Health Organisation. Mental health action plan 2013-2020. Geneva: World Health Organisation, 2013.
4. Walker IF, Stansfield J, Makurah L, *et al*. Delivering national public mental health – experience from England. *Journal of Public Mental Health*. 2019; 18(2):112-123.
5. Remes O, Lafortune L., Brayne C, *et al*. State-of-the-art review examining the key determinants of population mental health across the life course, as well as policy and practice guidance on this topic. PROSPERO 2019 CRD42019138753. Available from: http://www.crd.york.ac.uk/PROSPERO/display_record.php?ID=CRD42019138753

For peer review only - http://bmjopen.bmj.com/site/about/guidelines.xhtml

6. McManus S, Meltzer H, Brugha T, *et al*. Adult psychiatric morbidity in England, 2007. Results of a household survey. Health and Social Information Centre, Social Care Statistics, 2009.

7. Prince M, Patel V, Saxena S *et al*. No health without mental health. *Lancet*. 2009; 370:859-77.

8. Matheson FI, Smith KLW, Moineddin R, *et al*. Mental health status and gender as risk factors for onset of physical illness over 10 years. *Journal of Epidemiology & Community Health*. 2013; 68:64-70.

9. Gan Y, Gong Y, Tong X *et al*. Depression and the risk of coronary heart disease: a meta-analysis of prospective cohort studies. *BMC Psychiatry*. 2016; 14:371.

10. Russ TC, Stamatakis E, Hamer M, *et al*. Association between psychological distress and mortality: individual participant pooled analysis of 10 prospective cohort studies. *BMJ* 2012 Jul 31; 345:e4933.

11. McManus S, Meltzer H, Campion J. Cigarette smoking and mental health in England. Data from the Adult Psychiatric Morbidity Survey. London: National Centre for Social Research, 2010.

12. Walker ER, McGee RE, Druss BG. Mortality in mental disorders and global disease burden implications: a systematic review and meta-analysis. *JAMA Psychiatry*. 2015; 72: 334-41.

13. ONS Sickness absence in the labour market. Office for National Statistics. 2017. https://www.ons.gov.uk/employmentandlabourmarket/peopleinwork/labourproductivity/articles/sicknessabsenceinthelabourmarket/2016 (cited 9 December 2019).

14. McManus S, Bebbington P, Jenkins R *et al*. Mental health and wellbeing in England: Adult Psychiatric Morbidity Survey 2014. Leeds: NHS Digital, 2016.

For peer review only

15. Campion J. Public mental health: evidence practice and commissioning. Royal Society for Public Health, 2019.

16. Department of Health. No health without mental health: a cross-government mental health outcomes strategy for people of all ages, 2011.

17. Lyubomirsky S, King L, Diener E. The benefits of frequent positive affect: Does happiness lead to success? *Psychological Bulletin.* 2005a; 131(6):803-855.

18. Koivumaa-Honkanen H, Honkanen R, Viinamaeki H, *et al*. Life satisfaction and suicide: a 20-year follow-up study. *Am J Psychiatr*. 2001; 158:433-9.

19. Davidson KW, Mostofsky E, Whang W. Don't worry, be happy: positive affect and reduced 10-year incident coronary heart disease: the Canadian Nova Scotia Health Survey. *Eur Heart J*. 2010; 31(9):1065-70.

20. Chida Y, Steptoe A. Positive psychological wellbeing and mortality: a quantitative review of prospective observational studies. *Psychosomatic Medicine*. 2008; 70:741-756.

21. Lamers SMA, Bolier L, Westerhol GJ *et al*. The impact of emotional well-being on long-term recovery and survival in physical illness: a meta-analysis. *J Behav Med*. 2012; 35(5):538-547.

22. Kim ES, Kubzansky LD, Soo J *et al*. Maintaining healthy behavior: A prospective study of psychological well-being and physical activity. *Annals of Behavioral Medicine*. 2017; 51(3):337-347.

23. Tenney ER, Poole JM, Diener E. Does positivity enhance work performance: Why, when, and what we don't know? *Res. Organ. Behav*. 2016; 36:27-36.

24. Mental Health Taskforce. The Five Year Forward View for Mental Health, February 2016.

For peer review only - http://bmjopen.bmj.com/site/about/guidelines.xhtml

25. Cummins I. The impact of austerity on mental health service provision: A UK perspective. *Int J Environ Res Public Health.* 2018;15(6): 1145.

26. Mattheys K. The coalition, austerity and mental health. *Disability and Society*. 2015; 30(3), 475-478.

27. Better Mental Health for All: A public health approach to mental health improvement. London: Faculty of Public Health and Mental Health Foundation, 2016.

28. Burstow P. Newbigging K. Tew J. *et al*. Investing in a Resilient Generation: Keys to a Mentally Prosperous Nation. Birmingham: University of Birmingham, 2018.

29. Royal College of Psychiatrists position statement. No health without public mental health: the case for action. London: Royal College of Psychiatrists, 2010.

30. Moreno-Peral P, Conejo-Cerón S, Motrico E *et al*. Risk factors for the onset of panic and generalised anxiety disorders in the general adult population: a systematic review of cohort studies. *J Affect Disord*. 2014;168:337-48.

31. Richardson T, Elliott P, Roberts R. The relationship between personal unsecured debt and mental and physical health: A systematic review and meta-analysis. *Clinical Psychology Review*. 2013; 33(8):1148-1162.

32. Meltzer H, Bebbington P, Brugha T. The relationship between personal debt and specific common mental disorders. *Eur J Public Health*. 2013; 23(1):108-13.

33. Dijkstra-Kersten SMA, Biesheuvel-Leliefeld KEM, Wouden JC *et al*. Associations of financial strain and income with depressive and anxiety disorders. *J Epidemiol Community Health*. 2015; 69:660-665.

34. Howden-Chapman PL, Chandola T, Stafford M *et al*. The effect of housing on the mental health of older people: the impact of lifetime housing history in Whitehall II.

For peer review only - http://bmjopen.bmj.com/site/about/guidelines.xhtml

BMC Public Health. 2011;11:682 https://whatworkswellbeing.org/product/tackling-loneliness-full-review/

35. Jonas S, Khalifeh H, Bebbington PE *et al*. Gender differences in intimate partner violence and psychiatric disorders in England: results from the 2007 adult psychiatric morbidity survey. *Epidemiology and Psychiatric Sciences*. 2014; 23(2):189-99.
36. Teychenne M, Costigan SA, Parker K. The association between sedentary behaviour and risk of anxiety: a systematic review. *BMC Public Health*. 2015; 15:513.
37. Zhai L, Zhang Y, Zhang D. Sedentary behaviour and the risk of depression: a meta-analysis. *Br J Sports Med*. 2015; 49(11):705-9.
38. Harris J, Hall J, Meltzer H *et al*. Health, mental health and housing conditions in England. London: Eaga Charitable Trust and NatCen, 2010. http://natcen.ac.uk/media/23582/health-mental-health-housing.pdf.
39. Jones AD. Food insecurity and mental health status: A global analysis of 149 countries. *American Journal of Preventive Medicine*. 2017; 53(2):264-273.
40. Power M, Uphoff E, Kelly B *et al.* Food insecurity and mental health: an analysis of routine primary care data of pregnant women in the Born in Bradford cohort. *Journal of Epidemiology and Community Health.* 2017; 71(4):324-328.
41. Hodgson KJ, Shelton KH, van den Bree MBM. Mental health problems in young people with experiences of homelessness and the relationship with health service use: a follow-up study. *Evid Based Mental Health*. 2014; 17:76-80.
42. A guide to community-centred approaches for health and wellbeing. Full report. London: Public Health England, 2015.

For peer review only

For peer review only - http://bmjopen.bmj.com/site/about/guidelines.xhtml

43. Richardson, KM, Rothstein, HR.Effects of occupational stress management intervention programs: a meta-analysis. *Journal of Occupational Health Psychology*. 2008; Jan; 13(1) 69-93.

44. Goldie I, Grant S. Brighter Futures: Supporting mental health in later life. London: Mental Health Foundation, 2011. Available at: https://www.mentalhealth.org.uk/sites/default/files/Brighter_Futures_Report.pdf.

45. Mental health Foundation. Standing Together Project. 2016 Available at: https://www.mentalhealth.org.uk/projects/standing-together (cited 9 December 2019).

46. Woodhead C, Khondoker M, Lomas R, *et al*. Impact of co-located welfare advice in healthcare settings: Prospective quasi-experimental controlled study. *British Journal of Psychiatry*. 2017; 211(6), 388-395. doi:10.1192/bjp.bp.117.202713

47. Victor C. *et al* 2018. Current issues and debates in promoting physical activity among older people. Springer International Publishing.

48. Hoffman TC, Glasziou P., Boutron I, *et al*. Better reporting of interventions: template for intervention description and replication (TIDieR) checklist and guide. *BMJ*. 2014; 348:g1687 doi:10.1136/bmj.g1687.

For peer review only - http://bmjopen.bmj.com/site/about/guidelines.xhtml

BMJ Open

The delivery of community-centred public mental health interventions in diverse areas in England: a mapping study protocol.

Journal:	*BMJ Open*
Manuscript ID	bmjopen-2020-037631.R1
Article Type:	Protocol
Date Submitted by the Author:	04-Jun-2020
Complete List of Authors:	Duncan, Fiona; Durham University, Sport and Exercise Sciences<br>McGrath, Mike; UCL, Division of Psychiatry; UCL, Department of Primary Care and Population Health<br>Baskin, Cleo; Imperial College London, Department of Primary Care and Public Health<br>Osborn, David; UCL, Division of Psychiatry<br>Dykxhoorn, Jen; UCL, Division of Psychiatry; UCL, Department of Primary Care and Population Health<br>Kaner, Eileen; Newcastle University, Institute of Health and Society<br>Gnani, Shamini; Imperial College London, Department of Primary Care and Public Health<br>LaFortune, Louise; University of Cambridge, Institute of Public Health<br>Lee, Caroline; University of Cambridge, Institute of Public Health<br>Walters, Kate; University College London, Primary Care and Population Health<br>Kirkbride, James; University College London, Division of Psychiatry<br>Fischer, Laura; McPin Foundation<br>Jones, Oli; McPin Foundation<br>Pinfold, Vanessa; McPin Foundation,<br>Stansfield, J; Public Health England, Health Improvement Directorate<br>Oliver, Emily; Durham University, Department of Sport and Exercise Sciences
**Primary Subject Heading**:	Public health
Secondary Subject Heading:	Mental health, Health services research, Health policy
Keywords:	PUBLIC HEALTH, MENTAL HEALTH, Health policy < HEALTH SERVICES ADMINISTRATION & MANAGEMENT, Adult psychiatry < PSYCHIATRY

SCHOLARONE™
Manuscripts

I, the Submitting Author has the right to grant and does grant on behalf of all authors of the Work (as defined in the below author licence), an exclusive licence and/or a non-exclusive licence for contributions from authors who are: i) UK Crown employees; ii) where BMJ has agreed a CC-BY licence shall apply, and/or iii) in accordance with the terms applicable for US Federal Government officers or employees acting as part of their official duties; on a worldwide, perpetual, irrevocable, royalty-free basis to BMJ Publishing Group Ltd ("BMJ") its licensees and where the relevant Journal is co-owned by BMJ to the co-owners of the Journal, to publish the Work in this journal and any other BMJ products and to exploit all rights, as set out in our licence.

The Submitting Author accepts and understands that any supply made under these terms is made by BMJ to the Submitting Author unless you are acting as an employee on behalf of your employer or a postgraduate student of an affiliated institution which is paying any applicable article publishing charge ("APC") for Open Access articles. Where the Submitting Author wishes to make the Work available on an Open Access basis (and intends to pay the relevant APC), the terms of reuse of such Open Access shall be governed by a Creative Commons licence – details of these licences and which Creative Commons licence will apply to this Work are set out in our licence referred to above.

Other than as permitted in any relevant BMJ Author's Self Archiving Policies, I confirm this Work has not been accepted for publication elsewhere, is not being considered for publication elsewhere and does not duplicate material already published. I confirm all authors consent to publication of this Work and authorise the granting of this licence.

For peer review only

The delivery of community-centred public mental health interventions in diverse areas in England: a mapping study protocol.

Duncan F.[1], McGrath M.[2], Baskin C.[4], Osborn D.[2,7], Dykxhoorn J.,[2,3] Kaner E.[5], Gnani S.[4], Lafortune L.[6], Lee C.[6], Walters K.[3], Kirkbride J.B.[2], Fischer L.E.[8], Jones O.[8], Pinfold V.[8], Stansfield J.[9], Oliver E.[1], on behalf of the NIHR SPHR Public Mental Health Programme.

[1]Department of Sport and Exercise Sciences, Durham University, 42 Old Elvet, Durham, DH1 3HN,

[2]Division of Psychiatry, UCL, Maple House, 149 Tottenham Court Road, London, W1T 7BN.

[3]Department of Primary Care and Population Health, UCL, Royal Free, Rowland Hill Street London, NW3 2PF.

[4] Department of Primary Care and Public Health, School of Public Health, Imperial College London, Reynolds Building, St Dunstan's Road, London, W6 8RP.

[5]Institute of Health and Society, Baddiley-Clark Building, Newcastle University, Newcastle upon Tyne, NE2 4AX.

[6]Cambridge Institute of Public Health, Cambridge University, Forvie Site, Robinson Way, Cambridge, CB2 0SR.

[7]Camden and Islington NHS Foundation Trust, London, NW1 OPE

[8]McPin Foundation, 7-14 Great Dover Street, London, SE1 4YR

[9]Health Improvement Directorate, Public Health England.

Corresponding author: Fiona Duncan, Department of Sport and Exercise Sciences, Durham University, 42 Old Elvet, Durham, DH1 3HN, 0191 334 4676, fiona.h.duncan@durham.ac.uk

Keywords: public mental health, community-centred interventions, intervention mapping.

Word Count: 2607

For peer review only

For peer review only - http://bmjopen.bmj.com/site/about/guidelines.xhtml

Abstract

Background

Public Mental Health (PMH) is a global challenge and a UK priority area for action. However, to progress, practitioners require a stronger evidence base regarding the effectiveness of approaches, particularly regarding promotion and prevention through community-centred interventions. In addition, policy-makers need to understand what is being delivered, particularly in areas of high need, to identify promising practices or gaps in PMH provision. Thirdly, and importantly, the public need better information regarding what approaches and services are available to them. We report a protocol designed to 1) identify the types of community-centred interventions employed in purposively selected diverse geographical areas of England to improve PMH outcomes and 2) describe the type, target population, content and outcome measures of each intervention.

Methods and analysis

Five local authority areas of England were selected based on either high social deprivation or differing ethnic population statistics and geographical locations. Community-centred interventions in each area will be identified through: (i) desk-based data capture from standardised searches of publicly-available information (e.g. policy, strategy, intervention advertising), (ii) established professional networks and service contacts, (iii) chain-referral sampling of individuals involved in local MHP and prevention and (iv) peer researchers, who will use their personal experience and local knowledge to help identify potentially relevant organisations. Data of the key features of the interventions will be extracted from individuals either by structured interview or by electronic questionnaire with information regarding the intervention(s) of which they have knowledge. Initial data analysis will involve tabulating descriptive information; grouping interventions according to intervention type, target population, risk/protective factor and intended primary outcome. A descriptive comparison will be made between selected geographical areas.

For peer review only - http://bmjopen.bmj.com/site/about/guidelines.xhtml

Ethics and Dissemination

Ethical approval was obtained Durham University's Department of Sport and Exercise Sciences Research Ethics Committee. We plan to disseminate our findings at relevant conferences, meetings and through peer-reviewed journals. We also plan to disseminate to the public and intervention providers through social media and/or newsletters.

Article Summary

Strengths and limitations of this study

- Multiple sources are used to collect data.
- Protocol has been piloted in two areas and refined.
- A range of geographical areas of England have been included.
- It cannot be guaranteed that key individuals will be able to provide accurate information or that websites will be up-to-date.
- Only services that explicitly aim to improve the mental health of adults have been included (services aimed at only children and adolescents have been excluded).

For peer review only - http://bmjopen.bmj.com/site/about/guidelines.xhtml

For peer review only

Background

Public mental health (PMH) or population mental health is an emerging field and consequently there is still a lack of consensus regarding its definition and key components [1]. The World Health Organisation's (WHO) [2] PMH approach emphasises the distinction between mental health and mental illness, stating that mental health should be considered to be more than the absence of illness, and therefore conceptualisations of PMH should include mental well-being promotion as well as mental illness prevention. Mental well-being promotion involves encouraging good mental affect, positive feelings such as life satisfaction and happiness, reducing inequalities, building social capital, enhancing quality of life and enabling optimal psychological and psychophysiological development throughout the life course. Mental illness prevention involves reducing the incidence, prevalence and recurrence of mental health problems, as well as reducing the risk factors and the impact of mental illness on the affected person [2]. Consequently, WHO recommended in their mental health action plan several actions that focused on the promotion of positive mental health and emotional well-being as well as the prevention of mental health issues [3]. Consistent with this, Public Health England (PHE), a UK Government agency, also conceptualises PMH as consisting of the promotion of well-being and good mental affect, and the prevention of mental health problems and suicide [4]. A key aspect of PHE's approach to PMH is the recognition that mental health is a complex determinant of overall health and wellbeing and therefore actions that aim to minimise risk factors and enhance protective factors of mental health should be incorporated into a wide range of public health policies [4]. In line with the viewpoint of WHO and PHE, the conceptualisation of PMH that will be adopted for this protocol includes adverse and positive mental health [5].

Improving PMH is important as each year, in England, almost a quarter of adults experience at least one mental health problem [6] and 17% experience a sub-threshold mental health problem [6]. As well as the impact on the individual, this creates a substantial economic and social burden for the country as mental health problems have been reported to be associated with increased risk of physical

For peer review only - http://bmjopen.bmj.com/site/about/guidelines.xhtml

illness [7,8] such as coronary heart disease [9], increased mortality [10], higher rates of health risk behaviours such as smoking [11], reduced life expectancy [12], work absence [13], unemployment [14], homelessness [15] and reduced quality of life [16]. Conversely, good mental wellbeing has been reported to be associated with reduced mental health problems [17] and suicide [18], reduced heart disease [19], reduced mortality and increased life expectancy [20], improved recovery from physical illness [21], increased physical activity [22], reduced absenteeism [23] increased productivity at work, higher income and stronger social relationships [17].

Despite the high prevalence of mental health problems there has been a chronic underinvestment in mental health prevention across the English National Health Service (NHS) in recent years [24] and provision of interventions and services that support PMH in local areas has been badly affected by austerity measures introduced by the UK government in 2010 [25,26,27]. There is ongoing concern about how to promote PMH in areas experiencing deprivation, in particular [25,26,27].

A range of policy documents and publications have highlighted the importance of addressing the modifiable determinants of PMH within communities [15, 27, 28, 29]. These modifiable potential factors include both acute and chronic determinants such as economic disadvantage [6, 30], debt and financial difficulties [31,32,33,34], unemployment [14], social isolation and loneliness [14], intimate partner violence [35], sedentary lifestyles [36,37], fuel poverty [38], food insecurity [39,40], homelessness [41] and belonging to an ethnic minority group and other groups that experience stigma/marginalisation [14].

If causal, such a wide range of inter-related modifiable factors suggests that a multitude of approaches may be needed to improve mental health. However, the current evidence base for the effectiveness and cost-effectiveness of community-centred PMH interventions for adults is limited [42]. Promising examples of PMH interventions include workplace-based stress management interventions [43], community interventions for loneliness in older adults [44,45], and welfare benefits and debt advice interventions based in GP practices [46]. While these show promise to improve mental health and

For peer review only - http://bmjopen.bmj.com/site/about/guidelines.xhtml

wellbeing outcomes, the precise 'active ingredients' of these interventions need further evidence [47]. Further, in order to progress the provision of PMH services and approaches we need to understand what is currently being delivered in local areas, so that the assets and capabilities available in different communities addressing modifiable determinants of mental health are accurately mapped. Specifically, we need to identify any promising practice, learning from existing (successful and unsuccessful) practice, inconsistencies, variations or gaps in service delivery and what evidence exists concerning the effect they have on meeting population needs. As part of a larger National Institute of Health Research (NIHR) funded programme through the School for Public Health Research (SPHR), the present study protocol aims to:

1) identify the types of community-centred interventions employed in purposively selected diverse geographical areas of England to improve PMH outcomes and
2) describe the type, target population, risk/protective factor, content, reach, effectiveness, cost-effectiveness and intended outcome measures of each intervention.

Methods

Study Design

A mapping approach was undertaken to identify community-centred interventions across five selected areas of England.

Selected areas of England

The following local authority areas of England have been selected: Redcar and Cleveland (North East), Cambridgeshire and Peterborough (East), Blackburn (North West) and Camden and Islington, Hammersmith and Fulham (Greater London).

The process of selecting these areas was as follows: 1) a longlist of candidate local authority areas in the North East, North West, London and East of England was created to ensure that a wide geographic spread of areas were represented. 2) A range of statistics from the Office of National Statistics (ONS)

For peer review only - http://bmjopen.bmj.com/site/about/guidelines.xhtml

annual population survey regarding these local authority areas were examined, including statistics on deprivation. 3) The final areas were chosen as they have diverse social deprivation and/or ethnic populations, were proximal to research group institutions associated with the NIHR SPHR and therefore research group members could use established contacts with relevant local authority personnel to maximise response rates.

This protocol was piloted by mapping two of these selected areas (Redcar and Cleveland, Camden and Islington) between August 2019 and January 2020. We plan to have completed the mapping of the remaining areas by the end of May 2020.

Inclusion and Exclusion criteria

The following inclusion criteria will be applied to identify potential PMH interventions:

- Any intervention which explicitly aims to improve adult mental health and/or well-being. Such aims may be expressed as: to reduce social isolation, to improve emotional well-being, to increase happiness, to improve resilience, to decrease stress or to improve confidence and/or self-esteem. This aim does not have to be stated in materials that participants have access to for the intervention to be included, it could be stated verbally by an intervention provider. The intervention might consist of support, individual advice giving, signposting, activity groups, social groups or referral to community services. These programmes and interventions can be aimed at the general population or targeted at a specific sub-group i.e. older people, particular ethnic minorities, people who have job insecurity/debt/housing problems. Interventions delivered by individuals within the community (e.g. workshops, support groups) will be included.
- Any intervention which is delivered in, or available to residents of, the target local authority area, even if it is funded, delivered or managed within another local authority area or larger geographical area. Online and digital interventions will be included.

For peer review only - http://bmjopen.bmj.com/site/about/guidelines.xhtml

- Any intervention delivered by the government, the local authority, third sector organisations, social enterprises, the NHS (if non-clinical), or located in primary care settings (if non-clinical).
- Programmes and interventions that are available to local residents during the data collection period.

An intervention will be excluded if, it is:

- A clinical mental health intervention or an intervention that is primarily aimed at people identified with a diagnosed or suspected mental health condition.
- A wholly (for-profit) private sector funded intervention.
- An intervention which is exclusively aimed at children or adolescents, as this mapping exercise is focused on services for adults.
- Improving mental health and/or well-being is not one of the intervention's explicit aims (whether the aim is stated in writing or verbally by an intervention provider).

Data Collection

Two types of information will be sought during the mapping exercise: 1) identification of relevant interventions; 2) data on the key features of the identified services.

1) Identification of interventions. Much information about local services is unpublished and available information online can rapidly become out of date. Therefore, we will employ a range of approaches to create a list of interventions as exhaustive as possible, bringing together information from four sources: i) First of all desk-based internet searches of available information resources will be carried out. Local authority, relevant third sector, NHS and Clinical Commissioning Group websites will be searched for key policy documents, strategies, intervention advertising or guides to local services (Table 1). These sources will then be scrutinised for evidence of the existence of potentially relevant interventions. The following

For peer review only - http://bmjopen.bmj.com/site/about/guidelines.xhtml

key search terms will then be entered into an internet search engine: public mental health interventions, community development, community capacity building, community activities, anxiety/depression/stress courses/groups/workshops, wellbeing workshops/courses/groups/activities/sessions, life skills courses/classes, mental health promotion, mental health prevention, suicide prevention. Social media platforms will also be searched using these terms. Where available, the contact details of potentially relevant individuals and/or organisations identified through these searches of available information resources and these general internet searches will be noted and followed-up by email or by telephone for further information about interventions or services that they deliver. ii) Directly approaching by email and telephone potential key informants in each area and requesting information on interventions which fit our inclusion criteria (suggested key informants are presented in Table 1). Potential informants include relevant contacts from the public health department of local authorities, Clinical Commissioning Groups, voluntary sector provider organisations and signposting services (e.g. link workers or community navigators). The contact details of these informants will be obtained from the relevant organisation website or via previously established relationships between the research team and the organisation. iii) Chain-referral sampling (Snowballing). When individuals involved in local PMH promotion are contacted and respond with information for the study, they will be asked if they could provide details of any other public mental health interventions or relevant organisations that they are aware of in the specified local authority area. (iv) Peer researchers working within the mental health programme of the School for Public Health Research will use their personal experience of managing poor mental health and their local knowledge to assist in the identification of potentially relevant organisations or individuals. They will also contact these organisations to obtain information about services, and follow-up with services already identified to obtain further information.

For peer review only

For peer review only - http://bmjopen.bmj.com/site/about/guidelines.xhtml

Table 1 – Key policy, strategy and signposting documents from each selected area to be searched and examples of potential key local authority departments and organisations to be approached.

**Key Documents to be searched online**	**Key local authority departments to be contacted**	**Key Third Sector Organisations to be contacted**
• Public Mental Health strategy or action plan (e.g. prevention concordat, THRIVE, suicide prevention action plan)<br>• Clinical Commissioning Groups (CCG) Annual Report and Accounts<br>• Joint Health and Wellbeing Strategy<br>• Annual Public Health Report<br>• Joint Strategic Needs Assessment<br>• The Local Sustainability and Transformation Plan<br>• The Local Mental Health Strategy<br>• Signposting documents, websites and guides to mental health services.	• Public Health<br>• Housing<br>• Community Safety<br>• Community Health<br>• Social care<br>• Adult Education<br>• Libraries, arts and culture<br>• Leisure and recreation<br>• Neighbourhood and communities.	• MIND<br>• Citizens Advice Bureau<br>• Key Housing Associations<br>• Community Grants Organisation<br>• Job Centre Plus<br>• Age UK<br>• Age Concern<br>• Rethink Mental illness<br>• Samaritans<br>• Together<br>• CALM – Campaign against living miserably<br>• Carer's Trust

2) Data of the key features of the interventions. After identifying eligible interventions, we will extract information from websites, local authority documents and key individuals. The list of data to extract (Table 2) was adapted from the TIDieR checklist [48] to be applicable for complex public health interventions. Data will be extracted from individuals either by structured interview or an electronic questionnaire with information regarding the intervention(s) of which they have knowledge.

Table 2 – Data to be extracted about each intervention if available. (List adapted from the TIDieR checklist).

For peer review only

For peer review only - http://bmjopen.bmj.com/site/about/guidelines.xhtml

1.Name of Intervention

2.Type of Intervention (Befriending; practical advice, education and advocacy; counselling; peer support; strategy or policy; activity/social group).

3.Aims and Objectives (e.g. what problem or risk factor does the intervention seek to address)

4.What are the intended primary and secondary outcomes?

5.Description of the content of the intervention (what activities, procedures, processes take place, schedule, duration and intensity).

6.Target population (who is eligible to take part)

7.Geographical area of delivery

8.Setting (e.g. community centre, sports club, home-based)

9.What facilities, infrastructure and support are required to deliver intervention?

10.How is it delivered (online, one-to-one, in groups)?

11.Who delivers the intervention (expertise, background, training)?

12.Who is the provider?

13.How is it funded?

14.What is the funding period?

15.Has the intervention been evaluated?

16. If the intervention has been evaluated, are the following data available:

- What were the headline conclusions?
- What was the methodology?
- What were the participants characteristics, reach and uptake?
- What was the reported impact on outcomes, was there any feedback from service users?
- Were there any unintended consequences?
- Did people adhere to intervention
- Were these any issues with intervention fidelity (gaps in delivery, local adaptation, modification or personalisation)?
- Is the intervention sustainable or scalable?
- Is the intervention cost-effective?

Data Analysis

Descriptive information for each intervention will be tabulated. Interventions will be grouped according to their intervention type (examples of potential intervention types include signposting, befriending; legal or financial practical advice, education and advocacy; counselling, peer support, strategy and policy; activity/social group, green space/area regeneration), target population and

For peer review only - http://bmjopen.bmj.com/site/about/guidelines.xhtml

intended primary outcome (e.g. well-being or measures of mental distress). A descriptive comparison will be made between the selected geographical areas.

Patient and Public Involvement Statement

Peer researchers were recruited and coordinated by the McPin Foundation (a charity which aims to deliver more meaningful and more impactful patient and public involvement in every stage of mental health research). The peer research team was recruited to provide diverse perspectives based on their different localities, backgrounds, identities, and lived experiences of mental health challenges. The role of this team is to ensure that the research conducted in the public mental health programme is in line with the lived experience of mental health and is relevant to the challenges that people face in their daily lives.

The peer researchers have been involved with the piloting of the protocol by helping to identify potentially relevant organisations and individuals in two of the selected geographical areas that we intend to map and then contacting some of these organisations and individuals to gather information about PMH interventions they are delivering. We plan to have the peer researchers involved in a similar way when mapping the remaining selected geographical areas. They will also be involved in the analysis, write up and dissemination phases of the study. They will contribute to the writing of the study report and they will advise on the most effective approach of disseminating information to community groups, voluntary sector organisations, public health practitioners and other public stakeholders and will be involved in creating the content of the dissemination. This may include producing materials such as posters and leaflets, running workshops, using social media and their hashtag #IamPublicMentalHealth, and making vlogs or writing blogs.

Ethics and Dissemination

Ethical approval was obtained from Durham University's Department of Sport and Exercise Sciences Research Ethics Committee. Application Reference: SPORT-2019-06-28T15:10:42-lxkc61. We plan to disseminate our findings at relevant conferences, meetings and through peer-reviewed journals. We

For peer review only

For peer review only - http://bmjopen.bmj.com/site/about/guidelines.xhtml

will also disseminate our findings to the key personnel and organisations that participate in the study, to people who may benefit from identified interventions and the general public through social media, newsletters or local community groups. Finally, we will disseminate our findings through the SPHR's PMH virtual stakeholder network which consists of academics, voluntary sector individuals, public health practitioners, and public stakeholders.

Strengths and limitations of this study

Strengths

A major strength of this protocol is that it uses multiple sources to identify interventions and therefore increases the likelihood of all interventions in an area being discovered, including ones that the local authority or prominent third sector organisations are not aware of themselves. Another strength is that it has already been piloted in two of the selected areas, demonstrating its feasibility, and refinements have been made (e.g. we decided to only include interventions which were available during data collection period due to feasibility issues of collecting information about interventions that were no longer being delivered). It is also a strength that diverse geographical areas across are included in this study, increasing the generalisability of the mapping results.

Limitations

A potential weakness of the protocol is that there is no guarantee that key individuals who are approached for information will be willing or able to provide the most accurate and up-to-date information about interventions and services in their area. There is also the possibility that local authority and third sector websites may not be regularly updated or that there will be variations in their comprehensiveness. However, using more than one source to obtain information about each intervention should address these limitations. Two other potential limitations are that we are focused on interventions and services that are explicitly aiming to improve mental health and that we are excluding wholly private for-profit interventions. It is possible that many services are in existence that

For peer review only - http://bmjopen.bmj.com/site/about/guidelines.xhtml

do have a positive effect on participants' mental health but it is either not their explicit aim or they are a private enterprise e.g. a cycling group, a yoga course, a tennis club or a dance class. It should also be noted that there are other domains of well-being that are not represented in the design of this protocol. It is also a limitation that no areas in the South West of England are included in this study.

Declarations

Ethics approval and consent to participate

Ethical approval was obtained Durham University's Department of Sport and Exercise Sciences Research Ethics Committee. Application Reference: SPORT-2019-06-28T15:10:42-lxkc61

Availability of data and materials

Not applicable

Competing Interests

None

Funding

This project is funded by the National Institute for Health Research (NIHR) School for Public Health Research (SPHR) (Grant Reference Number PD-SPH-2015). The views expressed are those of the author(s) and not necessarily those of the NIHR or the Department of Health and Social Care.

Author Contributions

EO and DO are the Principal Investigators. JD is the Programme Manager. FD, MM, EO, SG, DO, KW, LL, JD, EK, LF, OJ, VP, JS were involved with designing the protocol methods. FD, MM, CB, CL. OJ and other local peer researchers will be involved with applying the protocol to collect data. JK, FD, MM, LF, VP and CB will be involved with data analysis. FD led on the writing and editing of the manuscript. All authors contributed to the writing and editing of the protocol for publication and read and approved the final manuscript.

Acknowledgements

We would like to sincerely thank all of the local authority and third sector staff and volunteers who gave up their time to contribute to this study. We would also like to thank our peer researchers and the McPin Foundation for their invaluable help.

DO is in part supported by the National Institute for Health Research (NIHR) Collaboration for Leadership in Applied Health Research and Care (CLAHRC) North Thames at Bart's Health NHS Trust and the University College London Hospitals National Institute for Health Research Biomedical Research Centre.

For peer review only - http://bmjopen.bmj.com/site/about/guidelines.xhtml

The NIHR School for Public Health Research is a partnership between the Universities of Sheffield; Bristol; Cambridge; Imperial; and University College London; The London School for Hygiene and Tropical Medicine (LSHTM); LiLaC – a collaboration between the Universities of Liverpool and Lancaster; and Fuse - The Centre for Translational Research in Public Health a collaboration between Newcastle, Durham, Northumbria, Sunderland and Teesside Universities

References

1. Davies SC, Mehta N. Public mental health: evidence to policy. *World Psychiatry*. 2015;14(1):44-45.
2. World Health Organisation. Prevention of mental disorders: effective interventions and policy options. Summary Report. Geneva: World Health Organisation, 2004.
3. World Health Organisation. Mental health action plan 2013-2020. Geneva: World Health Organisation, 2013.
4. Walker IF, Stansfield J, Makurah L, *et al*. Delivering national public mental health – experience from England. *Journal of Public Mental Health*. 2019; 18(2):112-123.
5. Remes O, Lafortune L., Brayne C, *et al*. State-of-the-art review examining the key determinants of population mental health across the life course, as well as policy and practice guidance on this topic. PROSPERO 2019 CRD42019138753. Available from: http://www.crd.york.ac.uk/PROSPERO/display_record.php?ID=CRD42019138753
6. McManus S, Meltzer H, Brugha T, *et al*. Adult psychiatric morbidity in England, 2007. Results of a household survey. Health and Social Information Centre, Social Care Statistics, 2009.
7. Prince M, Patel V, Saxena S *et al*. No health without mental health. *Lancet*. 2009; 370:859-77.

For peer review only - http://bmjopen.bmj.com/site/about/guidelines.xhtml

8. Matheson FI, Smith KLW, Moineddin R, *et al*. Mental health status and gender as risk factors for onset of physical illness over 10 years. *Journal of Epidemiology & Community Health.* 2013; 68:64-70.

9. Gan Y, Gong Y, Tong X *et al*. Depression and the risk of coronary heart disease: a meta-analysis of prospective cohort studies. *BMC Psychiatry*. 2016; 14:371.

10. Russ TC, Stamatakis E, Hamer M, *et al*. Association between psychological distress and mortality: individual participant pooled analysis of 10 prospective cohort studies. *BMJ* 2012 Jul 31; 345:e4933.

11. McManus S, Meltzer H, Campion J. Cigarette smoking and mental health in England. Data from the Adult Psychiatric Morbidity Survey. London: National Centre for Social Research, 2010.

12. Walker ER, McGee RE, Druss BG. Mortality in mental disorders and global disease burden implications: a systematic review and meta-analysis. *JAMA Psychiatry*. 2015; 72: 334-41.

13. ONS Sickness absence in the labour market. Office for National Statistics. 2017. https://www.ons.gov.uk/employmentandlabourmarket/peopleinwork/labourproductivity/articles/sicknessabsenceinthelabourmarket/2016 (cited 9 December 2019).

14. McManus S, Bebbington P, Jenkins R *et al*. Mental health and wellbeing in England: Adult Psychiatric Morbidity Survey 2014. Leeds: NHS Digital, 2016.

15. Campion J. Public mental health: evidence practice and commissioning. Royal Society for Public Health, 2019.

16. Department of Health. No health without mental health: a cross-government mental health outcomes strategy for people of all ages, 2011.

17. Lyubomirsky S, King L, Diener E. The benefits of frequent positive affect: Does happiness lead to success? *Psychological Bulletin.* 2005a; 131(6):803-855.

For peer review only - http://bmjopen.bmj.com/site/about/guidelines.xhtml

18. Koivumaa-Honkanen H, Honkanen R, Viinamaeki H, *et al*. Life satisfaction and suicide: a 20-year follow-up study. *Am J Psychiatr*. 2001; 158:433-9.

19. Davidson KW, Mostofsky E, Whang W. Don't worry, be happy: positive affect and reduced 10-year incident coronary heart disease: the Canadian Nova Scotia Health Survey. *Eur Heart J*. 2010; 31(9):1065-70.

20. Chida Y, Steptoe A. Positive psychological wellbeing and mortality: a quantitative review of prospective observational studies. *Psychosomatic Medicine*. 2008; 70:741-756.

21. Lamers SMA, Bolier L, Westerhol GJ *et al*. The impact of emotional well-being on long-term recovery and survival in physical illness: a meta-analysis. *J Behav Med*. 2012; 35(5):538-547.

22. Kim ES, Kubzansky LD, Soo J *et al*. Maintaining healthy behavior: A prospective study of psychological well-being and physical activity. *Annals of Behavioral Medicine*. 2017; 51(3):337-347.

23. Tenney ER, Poole JM, Diener E. Does positivity enhance work performance: Why, when, and what we don't know? *Res. Organ. Behav*. 2016; 36:27-36.

24. Mental Health Taskforce. The Five Year Forward View for Mental Health, February 2016.

25. Cummins I. The impact of austerity on mental health service provision: A UK perspective. *Int J Environ Res Public Health.* 2018;15(6): 1145.

26. Mattheys K. The coalition, austerity and mental health. *Disability and Society*. 2015; 30(3), 475-478.

27. Better Mental Health for All: A public health approach to mental health improvement. London: Faculty of Public Health and Mental Health Foundation, 2016.

For peer review only

28. Burstow P. Newbigging K. Tew J. *et al*. Investing in a Resilient Generation: Keys to a Mentally Prosperous Nation. Birmingham: University of Birmingham, 2018.

29. Royal College of Psychiatrists position statement. No health without public mental health: the case for action. London: Royal College of Psychiatrists, 2010.

30. Moreno-Peral P, Conejo-Cerón S, Motrico E *et al*. Risk factors for the onset of panic and generalised anxiety disorders in the general adult population: a systematic review of cohort studies. *J Affect Disord*. 2014;168:337-48.

31. Richardson T, Elliott P, Roberts R. The relationship between personal unsecured debt and mental and physical health: A systematic review and meta-analysis. *Clinical Psychology Review*. 2013; 33(8):1148-1162.

32. Meltzer H, Bebbington P, Brugha T. The relationship between personal debt and specific common mental disorders. *Eur J Public Health*. 2013; 23(1):108-13.

33. Dijkstra-Kersten SMA, Biesheuvel-Leliefeld KEM, Wouden JC *et al*. Associations of financial strain and income with depressive and anxiety disorders. *J Epidemiol Community Health*. 2015; 69:660-665.

34. Howden-Chapman PL, Chandola T, Stafford M *et al*. The effect of housing on the mental health of older people: the impact of lifetime housing history in Whitehall II. *BMC Public Health*. 2011;11:682 https://whatworkswellbeing.org/product/tackling-loneliness-full-review/

35. Jonas S, Khalifeh H, Bebbington PE *et al*. Gender differences in intimate partner violence and psychiatric disorders in England: results from the 2007 adult psychiatric morbidity survey. *Epidemiology and Psychiatric Sciences*. 2014; 23(2):189-99.

For peer review only - http://bmjopen.bmj.com/site/about/guidelines.xhtml

36. Teychenne M, Costigan SA, Parker K. The association between sedentary behaviour and risk of anxiety: a systematic review. *BMC Public Health*. 2015; 15:513.

37. Zhai L, Zhang Y, Zhang D. Sedentary behaviour and the risk of depression: a meta-analysis. *Br J Sports Med*. 2015; 49(11):705-9.

38. Harris J, Hall J, Meltzer H *et al*. Health, mental health and housing conditions in England. London: Eaga Charitable Trust and NatCen, 2010. http://natcen.ac.uk/media/23582/health-mental-health-housing.pdf.

39. Jones AD. Food insecurity and mental health status: A global analysis of 149 countries. *American Journal of Preventive Medicine*. 2017; 53(2):264-273.

40. Power M, Uphoff E, Kelly B *et al.* Food insecurity and mental health: an analysis of routine primary care data of pregnant women in the Born in Bradford cohort. *Journal of Epidemiology and Community Health.* 2017; 71(4):324-328.

41. Hodgson KJ, Shelton KH, van den Bree MBM. Mental health problems in young people with experiences of homelessness and the relationship with health service use: a follow-up study. *Evid Based Mental Health*. 2014; 17:76-80.

42. A guide to community-centred approaches for health and wellbeing. Full report. London: Public Health England, 2015.

43. Richardson, KM, Rothstein, HR.Effects of occupational stress management intervention programs: a meta-analysis. *Journal of Occupational Health Psychology*. 2008; Jan; 13(1) 69-93.

44. Goldie I, Grant S. Brighter Futures: Supporting mental health in later life. London: Mental Health Foundation, 2011. Available at: https://www.mentalhealth.org.uk/sites/default/files/Brighter_Futures_Report.pdf.

For peer review only - http://bmjopen.bmj.com/site/about/guidelines.xhtml

45. Mental health Foundation. Standing Together Project. 2016 Available at: https://www.mentalhealth.org.uk/projects/standing-together (cited 9 December 2019).

46. Woodhead C, Khondoker M, Lomas R, *et al*. Impact of co-located welfare advice in healthcare settings: Prospective quasi-experimental controlled study. *British Journal of Psychiatry*. 2017; 211(6), 388-395. doi:10.1192/bjp.bp.117.202713

47. Victor C. *et al* 2018. Current issues and debates in promoting physical activity among older people. Springer International Publishing.

48. Hoffman TC, Glasziou P., Boutron I, *et al*. Better reporting of interventions: template for intervention description and replication (TIDieR) checklist and guide. *BMJ*. 2014; 348:g1687 doi:10.1136/bmj.g1687.

For peer review only - http://bmjopen.bmj.com/site/about/guidelines.xhtml
